# 肺癌小分子靶向药物临床合理用药专家共识

**DOI:** 10.3779/j.issn.1009-3419.2025.106.10

**Published:** 2025-04-20

**Authors:** Liyan ZHAO, Kejing TANG, Expert Consensus Working Group on Rational Use and Monitoring of Small Molecule Targeted Drugs for Lung Cancer, Lung Cancer Professional Committee of Guangdong Medical Women’s Association

**Keywords:** 肺肿瘤, 小分子靶向药物, 合理用药, 疗效监测, 不良反应监测, Lung neoplasms, Small molecule targeted therapeutic drugs, Rational use of drugs, Efficacy monitoring, Adverse drug reaction monitoring

## Abstract

肺癌小分子靶向药物的临床应用显著改善了肺癌患者的生存期，但此类药物种类多、新药研发上市快、不良反应复杂且多为居家用药，增加了其不合理使用风险，同时疗效、安全性方面也容易出现监测不足的情况，最终影响治疗结局。本共识针对国内外上市的43种肺癌小分子靶向药物（或药物组合），为临床提供合理用药和疗效/不良反应监测的规范化建议，内容包括药物选择、剂量调整、疗效监测、不良反应监测、提高患者依从性等内容。本共识旨在提高肺癌小分子靶向药物合理用药水平和疗效/安全性监测质量，保障药物有效性与安全性，延长肺癌患者生存期，提升肺癌患者生存质量。

## Abstract (en)

The application of small molecule targeted drugs for lung cancer has significantly improved the survival of lung cancer patients. However, these drugs have a wide variety of types, fast development and market launch of new drugs, complex adverse reactions, and are mostly used at home, which increases the risk of irrational drug use. At the same time, insufficient monitoring of efficacy and safety is also prone to occur, ultimately affecting treatment outcomes. This consensus focuses on 43 small molecule targeted drugs or combinations for lung cancer, providing standardized recommendations for rational drug use and monitoring of efficacy/adverse reactions in clinical practice. The recommendations are regarding drug selection, dosage adjustment, efficacy monitoring, adverse reaction monitoring, and improvement of patient compliance. This consensus aims to improve the rational use and efficacy/safety monitoring quality of small molecule targeted drugs for lung cancer, ensure the effectiveness and safety of drug treatment, prolong the survival of lung cancer patients and improve their quality of life.

世界卫生组织国际癌症研究机构（International Agency for Research on Cancer, IARC）发布的数据^[1]^显示，肺癌是全球发病率和死亡率最高的恶性肿瘤，2022年全球肺癌新发病例为248.1万例，占新发肿瘤病例数的12.4%；死亡病例181.7万例，占肿瘤死亡病例数的18.7%。其中，我国肺癌发病率和死亡率亦均居恶性肿瘤首位，新发病例为106.1万例，死亡病例为73.3万例^[2]^。绝大部分肺癌患者的病理分型为非小细胞肺癌（non-small cell lung cancer, NSCLC），约占肺癌总人数的85%^[3]^，治疗方法包括手术治疗、放疗、化疗、靶向治疗、免疫治疗以及综合治疗等，其中小分子靶向药物在驱动基因阳性NSCLC患者的治疗中占据重要地位。肺癌小分子靶向药物具有疗效显著、给药方便、医疗资源消耗较少、对患者日常生活影响小等多方面优势，但此类药物种类繁多、研发上市速度快、不良反应复杂、院外用药普遍，增加了临床不合理用药风险。因此，由广东省女医师协会肺癌专业委员会牵头，组织肺癌领域的临床和药学专家，就国内外上市的肺癌小分子靶向药物的药物选择、剂量调整、疗效监测和不良反应监测等内容形成规范化共识，以期为临床合理用药和疗效/不良反应监测工作提供参考。

## 1 方法学

检索美国国立综合癌症网络（National Comprehensive Cancer Network, NCCN）指南、中国临床肿瘤学会（Chinese Society of Clinical Oncology, CSCO）指南、国家药品监督管理局（National Medical Products Administration, NMPA）药品说明书、美国食品药品监督管理局（Food and Drug Administration, FDA）药品说明书、Micromedex、PubMed、Clinicaltrials.gov等数据库。在共识讨论会议上，编写组中的临床专家和药学专家审阅和评估了小分子靶向药物合理用药相关的重要证据，基于证据级别和专家意见，最终形成9条推荐意见和8份用药指引附表，附表涉及肺癌小分子靶向药物在肺癌领域的应用范围、特殊人群用药、药代动力学特点、药物相互作用、用法用量及药物不良反应监测路径等。本共识推荐意见的级别和各级别所代表意义见表1。

**表1:** 共识推荐类别说明

各推荐类别的含义
1类：根据高质量临床证据，专家意见达成高度一致
2A类：根据低质量临床证据，专家意见达成高度一致；或根据高质量证 据，专家意见达成基本一致
2B类：根据低质量证据，专家意见达成基本一致
3类：不论根据何种质量临床证据，专家意见存在明显分歧

## 2 肺癌小分子靶向药物的合理使用

### 2.1 肺癌小分子靶向药物的选择

#### 2.1.1 肺癌小分子靶向药物的临床应用范围

肺癌驱动基因突变和相应小分子靶向药物的临床应用主要集中在NSCLC患者。亚洲人群和西方人群NSCLC驱动基因突变类型及突变率如图1所示^[4]^，包括表皮生长因子受体（epidermal growth factor receptor, EGFR）19号外显子缺失突变、21号外显子L858R突变、20号外显子插入突变、其他少见EGFR突变；间变性淋巴瘤激酶（anaplastic lymphoma kinase, ALK）重排；ROS原癌基因1（Ros proto-oncogene 1, ROS1）重排；B-Raf原癌基因（B-Raf proto-oncogene, BRAF）V600E突变；神经营养酪氨酸受体激酶（neurotrophic tyrosine receptor kinase, NTRK）融合；间质-上皮转化因子（mesenchymal-epithelial transition factor, MET）14号外显子跳跃突变；转染重排（rearranged during transfection, RET）重排；Kirsten大鼠肉瘤病毒癌基因同源物（Kirsten rat sarcoma viral oncogene homolog, KRAS）G12C突变、其他KRAS突变；人表皮生长因子受体2（human epidermal growth factor receptor 2, HER2）20号外显子插入突变；以及尚无靶向药物治疗的突变。

**图1:**
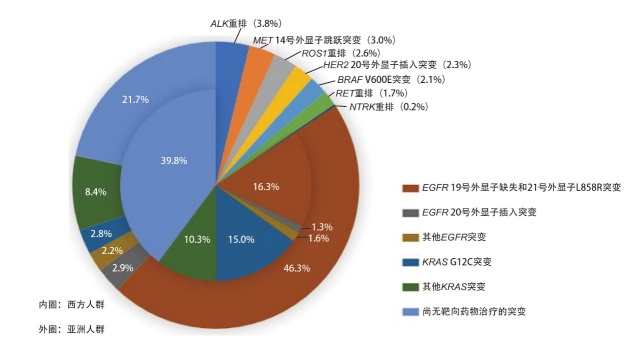
NSCLC患者肿瘤驱动基因突变率 NSCLC：非小细胞肺癌；EGFR：表皮生长因子受体；ALK：间变性淋巴瘤激酶；ROS1：ROS原癌基因1；BRAF：B-Raf原癌基因；NTRK：神经营养酪氨酸受体激酶；MET：间质-上皮转化因子；RET：转染重排基因；KRAS：Kirsten大鼠肉瘤病毒癌基因同源物；HER2：人表皮生长因子受体2。

截至2025年1月，NCCN和CSCO发布的非小细胞肺癌指南共涉及43种小分子靶向药物（或药物组合）用于肺癌治疗，包括吉非替尼、厄洛替尼、埃克替尼、阿法替尼、达可替尼、奥希替尼、阿美替尼、伏美替尼、贝福替尼、瑞齐替尼、瑞厄替尼、Lazertinib（与埃万妥单抗联用）、舒沃替尼、克唑替尼、阿来替尼、塞瑞替尼、恩沙替尼、布格替尼、伊鲁阿克、依奉阿克、洛拉替尼、恩曲替尼、安奈克替尼、瑞普替尼、达拉非尼+曲美替尼、达拉非尼、维莫非尼、Encorafenib+Binimetinib、拉罗替尼、赛沃替尼、谷美替尼、伯瑞替尼、特泊替尼、卡马替尼、普拉替尼、塞普替尼、Cabozantinib、氟泽雷塞、格索雷塞、Sotorasib、Adagrasib、吡咯替尼和安罗替尼。

由于抗肿瘤药物的研发和临床实践发展迅速，NMPA适应证可能滞后于临床实践，因此本共识进一步纳入FDA批准的适应证、NCCN指南（NSCLC 2025.V1版）、《CSCO非小细胞肺癌诊疗指南2024》、《新型抗肿瘤药物临床应用指导原则（2024版）》作为证据来源，经过筛选整合，得出各肺癌小分子靶向药物在肺癌领域的推荐应用范围。对于驱动基因阳性的局部晚期或转移性NSCLC患者小分子靶向药物的快速选择可参考图2。

**图2:**
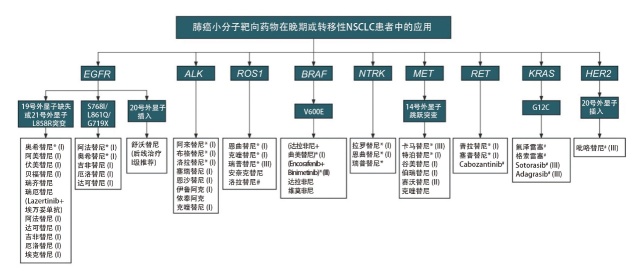
基于驱动基因突变的肺癌小分子靶向药物临床应用 *：NCCN NSCLC指南（2025.V1）优先推荐；^#^：仅适用于后线治疗；I、II、III：《CSCO非小细胞肺癌诊疗指南2024》中的推荐等级。CSCO：中国临床肿瘤学会；NCCN：美国国立综合癌症网络。

推荐意见1：选用肺癌小分子靶向药物时，应根据各靶向药物在肺癌领域的应用范围进行（附表1，http://www.lungca.org/files/2024s64-s1.pdf）。对于驱动基因阳性的局部晚期或转移性NSCLC患者，小分子靶向药物的快速选择可参考图2。（推荐级别：1类）

推荐意见2：本共识中部分药物的应用范围尚未获NMPA批准，推荐基于FDA说明书和权威指南，医疗机构应协助做好超说明书用药备案工作，并做好超说明书用药患者知情同意。（推荐级别：2A类）

#### 2.1.2 特殊人群药物选择

肺癌患者可能合并特殊的生理或病理状态，如肝肾功能不全、心脏疾病、有备孕或哺乳需求、妊娠等。对于特殊患者人群，应尽量选择已有该人群研究数据和用法用量指导的药物，并兼顾药物不良反应（adverse drug reaction, ADR）特点和药物相互作用（drug-drug interaction, DDI），降低潜在用药安全风险。肝肾功能不全患者是否可以服用肺癌小分子靶向药物参考附表2（http://www.lungca.org/files/2024s64-s2.pdf），对于尚无资料的重度肝肾功能不全人群，说明书未予禁用的，应根据药代动力学特点（附表3，http://www.lungca.org/files/2024s64-s3.pdf）慎用，并加强不良反应监测。有心脏基础疾病的患者用药时，应权衡利弊，尽量选用心脏毒性发生率较低的靶向药物，肺癌小分子靶向药物的心脏毒性种类及发生率见附表4（http://www.lungca.org/files/2024s64-s4.pdf）。对于妊娠期患者，有限的临床研究结果显示部分小分子靶向药物的抗肿瘤疗效和耐受性似乎与普通人群相似^[5-14]^，但这些研究样本量较少，尚不足以支持靶向药物在妊娠期患者中的使用，建议育龄女性在服用此类药物期间避免妊娠，停药后按照附表5（http://www.lungca.org/files/2024s64-s5.pdf）建议的间隔时间避孕。用药期间避免哺乳，停药后参考附表5中的间隔时间开始哺乳。各靶向药物ADR特点详见本文“3.2 ADR监测”，DDI信息见附表6（http://www.lungca.org/files/2024s64-s6.pdf）。

推荐意见3：肝肾功能不全患者应尽量选择已有特殊人群研究数据和用法用量指导的药物（附表2），对于尚无用药安全性数据的肝肾功能不全人群，说明书未予禁用的，应根据药代动力学数据（附表3）慎用，并加强不良反应监测。（推荐级别：2A类）

推荐意见4：药物选择时应充分考虑ADR特点，特别是致命不良反应如心脏毒性（附表4），以及DDI（附表6），同时兼顾患者经济水平和药品可及性。（推荐级别：1类）

推荐意见5：建议育龄女性在服用此类药物期间避免妊娠和哺乳，并按照附表5停药后所需避孕时间进行避孕。（推荐级别：2B类）

### 2.2 肺癌小分子靶向药物的用法用量

#### 2.2.1 一般用法用量

肺癌小分子靶向药物均为口服给药。赛沃替尼、塞普替尼需按体重范围确定给药剂量，其他大部分药物无需根据体重计算剂量。贝福替尼、布格替尼、伊鲁阿克、瑞普替尼应低剂量起始，如患者可耐受再增加至目标剂量。对于不同突变类型，埃克替尼有不同的推荐剂量。Cabozantinib在儿童或与纳武利尤单抗合用时采用更低剂量。除安罗替尼连用2周、停药1周外，其他药物均为连续服药。各靶向药物的给药剂量按照附表7（http://www.lungca.org/files/2024s64-s7.pdf）执行，此外，是否可与食物同服、漏服补服方案以及部分药物的管饲给药方案亦参考附表7。

#### 2.2.2 给药剂量调整

##### 2.2.2.1 肝肾功能不全患者的剂量调整

根据原研药品说明书、Micromedex数据库及文献证据，对肝肾功能不全患者的靶向药物剂量调整方案作出指引（附表2）。

##### 2.2.2.2 ADR致剂量调整

ADR致肺癌小分子靶向药物的剂量调整较为常见，根据不良事件通用术语标准（Common Terminology Criteria for Adverse Events, CTCAE）5.0进行ADR分级，针对不同ADR类型和严重程度，各靶向药物说明书均列有具体的减量标准和减量梯度（附表7）。在ADR致剂量调整这项工作中，对ADR的监测尤为重要，及时发现和评估ADR，采取相应的减量、停药等措施，是保障患者用药安全、延长用药时间的关键。

##### 2.2.2.3 DDI致剂量调整

当拟接受肺癌小分子靶向药物治疗的患者存在合用药物时，应充分评估靶向药物是否可以使用，如非完全禁止合用的情况，应进一步评估是否需要调整剂量。吉非替尼、厄洛替尼、奥希替尼、拉罗替尼、Cabozantinib与CYP3A4强诱导剂合用时需调整剂量；舒沃替尼、克唑替尼、塞普替尼、布格替尼、洛拉替尼、恩曲替尼、Encorafenib、拉罗替尼、塞普替尼、Cabozantinib与CYP3A/CYP3A4的强抑制剂合用时需调整剂量；阿法替尼与P-gp抑制剂或P-gp诱导剂合用，布格替尼与CYP3A中效抑制剂及中效诱导剂合用，洛拉替尼与CYP3A中效诱导剂、氟康唑合用，普拉替尼与P-gp和强效CYP3A共同抑制剂合用时需调整剂量；克唑替尼与CYP3A底物合用、伯瑞替尼与MATE2-K底物合用、特泊替尼与P-gp底物合用时需调整底物剂量。具体DDI情况下的剂量调整参考附表6。

##### 2.2.2.4 基于治疗药物监测（therapeutic drug monitoring, TDM）的剂量调整

多数肺癌靶向药物的暴露量与反应（exposure-response, E-R）关系尚不明确，根据FDA批准文件中Pharmacology and Biopharmaceutics Review的临床药理数据以及文献研究进展，暴露量和疗效可能有关的药物有克唑替尼^[15]^、阿来替尼^[15,16]^、布格替尼^[17]^、维莫非尼^[18-21]^，暴露量与不良反应可能有关的药物有吉非替尼^[22,23]^、厄洛替尼^[24-28]^、阿法替尼^[29-32]^、达可替尼、奥希替尼^[33-35]^、塞瑞替尼^[36]^、布格替尼、洛拉替尼^[37]^、维莫非尼、卡马替尼、普拉替尼、Cabozantinib^[38]^。这些研究的样本量一般较小，且同一药物不同研究的方案设计不同，其得出的结论也常有不一致，随着临床研究的进一步开展可能得出新的结论。因此基于TDM的肺癌小分子靶向药物的剂量调整证据尚不成熟，尚不推荐常规进行TDM以调整剂量。

推荐意见6：根据患者驱动基因突变类型、年龄、体重、器官功能、DDI、ADR等个体化数据，确定和调整肺癌小分子靶向药物的给药剂量。（推荐级别：1类）

推荐意见7：医疗机构可基于研究目的开展肺癌小分子靶向药物TDM，但不建议常规开展TDM用于剂量调整。当患者出现疗效不佳或不良反应时，医务人员可通过TDM结果和最新研究进展协助调整给药剂量。（推荐级别：2B类）

## 3 肺癌小分子靶向药物疗效及安全性监测

### 3.1 疗效监测

启用小分子靶向药物之前，应遵循实体瘤疗效评价标准（Response Evaluation Criteria in Solid Tumors, RECIST）1.1进行疗效相关基线评估，并在治疗过程中制定随访计划，定期评估，如有肿瘤快速进展症状可提前进行疗效评估。

### 3.2 ADR监测

#### 3.2.1 常见肺癌小分子靶向药物ADR特点及处理

肺癌小分子靶向药物所致ADR与传统化疗、免疫治疗等存在明显不同，常见毒性包括皮肤黏膜毒性、胃肠道毒性、药物性肝损伤、心脏毒性、间质性肺病（interstitial lung disease, ILD）等。如监护不足或处理不当可致患者依从性不佳、非必要停药甚至严重ADR的发生，严重影响肺癌治疗效果。本部分描述了肺癌小分子靶向药物ADR的种类、发生率、临床表现、评估及处理方法。

##### 3.2.1.1 皮肤黏膜相关ADR

皮肤黏膜相关ADR为小分子靶向药物常见ADR，包括皮疹、甲沟炎、口腔黏膜炎等。一项针对6种EGFR-酪氨酸激酶抑制剂（tyrosine kinase inhibitors, TKIs）ADR的网状meta分析研究^[39]^结果显示，皮疹发生率方面，阿法替尼为81.4%、厄洛替尼为64.1%、吉非替尼为56.9%、奥希替尼为47.7%、达可替尼为28.1%、埃克替尼为27.4%。多数ALK-TKIs（克唑替尼、塞瑞替尼、阿来替尼、布格替尼、洛拉替尼）皮疹发生率相对较低，仅恩沙替尼发生率较高（70.6%），其中严重皮疹发生率为11.2%^[40]^。甲沟炎发生率方面，阿法替尼、达可替尼发生率较高（分别为38.9%和36.2%），奥希替尼为26.3%、吉非替尼为16.2%、厄洛替尼为5.1%^[39]^。口腔黏膜炎方面，EGFR-TKIs发生率相对较高，阿法替尼为54.2%、达可替尼为37.2%、奥希替尼为22.8%、吉非替尼为19.1%；ALK-TKIs中克唑替尼致口腔黏膜炎发生率为12.4%，其他ALK-TKIs临床试验中未报道该项不良反应。

皮疹多在靶向治疗1-2周后发生，形态单一，以丘疹、脓疱疹为主，常发生于皮脂腺丰富的部位，严重时下肢亦可受累甚至遍及全身，多伴有瘙痒和皮肤干燥^[41,42]^。甲沟炎多发生于小分子靶向药物开始治疗的4-8周后，可发生于任何指（趾）甲，通常由根部的边缘开始出现红肿、疼痛，随后逐渐向两侧甲沟发展，表现为发炎、溃疡、出现化脓性肉芽组织等症状，使指（趾）甲内嵌^[43]^。口腔黏膜炎常在小分子靶向药物用药后13-19天出现，表现为口腔黏膜出现红斑、水肿或糜烂，进一步形成点状、片状溃疡，可波及上下唇、双颊、舌和口底黏膜，黏膜溃疡表面覆有伪膜、渗血，可引起疼痛、吞咽困难、味觉异常等^[41]^。肺癌小分子靶向药物可致严重皮肤ADR，例如中毒性表皮坏死松懈症、大疱性皮炎、Stevens-Johnson综合症和多形性红斑，需要引起临床重视。

对于皮肤黏膜相关ADR的处理，中国抗癌协会发布了《EGFR-TKI不良反应管理专家共识》^[44]^，根据共识建议，首先按照CTCAE 5.0标准对ADR进行评估分级，针对性选择处理方案。皮疹对症治疗药物主要有抗生素、抗组胺药、糖皮质激素，顽固性瘙痒可酌情使用加巴喷丁或普瑞巴林等药物；甲沟炎对症治疗药物主要有抗生素、抗真菌药物、糖皮质激素、碘酊等；对于口腔黏膜炎应根据ADR严重程度和可能的感染病原体应用局麻药、抗生素、抗真菌药、抗病毒药、全身镇痛药和抗焦虑药等。一般如出现3级及以上ADR，需按说明书调整靶向药物剂量。

##### 3.2.1.2 胃肠道ADR

肺癌小分子靶向药物的胃肠道ADR主要有腹泻、呕吐、便秘，其中腹泻最为常见。腹泻方面，一项针对6种EGFR-TKIs的网状meta分析^[39]^结果显示，奥希替尼发生率为48.1%、达可替尼为77.6%、阿法替尼为85.9%、厄洛替尼为34.8%、吉非替尼为37.5%、埃克替尼为12.8%。一项针对6种ALK-TKIs的网状meta分析^[40]^结果显示克唑替尼腹泻发生率为56.9%、阿来替尼为10.4%、塞瑞替尼为79.9%、布格替尼为44.3%、洛拉替尼为21.5%，恩沙替尼尚无腹泻数据。此外，Cabozantinib、依奉阿克致腹泻发生率相对较高，在临床试验中有50%以上的报道^[45]^。腹泻的处理参考国内已发布的EGFR、ALK抑制剂不良反应管理专家共识^[44,46]^，以及发表于ESMO Open的ALK-TKIs ADR管理专家共识^[47]^，对症治疗的常用药物主要有洛哌丁胺、益生菌、蒙脱石散。

部分ALK-TKIs致呕吐发生率相对较高，根据一项网状meta分析^[40]^的结果，呕吐发生率方面，塞瑞替尼、阿来替尼、克唑替尼发生率较高，分别为60.9%、48.7%和42.7%；布格替尼、恩沙替尼和洛拉替尼发生率相对较低，分别为20.33%、16.1%和12.8%。而EGFR-TKIs致呕吐的发生率相对较低，一般在10%以下^[39]^。此外，赛沃替尼、Cabozantinib^[45]^、依奉阿克、安奈克替尼、Encorafenib联合Binimetinib致呕吐发生率也超过30%。对于1级呕吐，维持给药剂量，并调整止吐药治疗方案；对于2级呕吐，停药至呕吐缓解至≤1级，再以原剂量重启；对于持续时间较长的2级呕吐和≥3级呕吐，停药至呕吐症状缓解至≤1级，再以降低药物剂量重启。同时注意部分药品说明书中未建议2级呕吐时停药，如赛沃替尼NMPA说明书未建议2级呕吐暂停服药。止呕药物方案可参考NCCN止呕指南（2023.V2），可选择的药物有5-HT_3_受体拮抗剂、地塞米松、神经激肽（neurokinin, NK）-1受体拮抗剂、抗焦虑药物、抑酸剂、氟哌啶醇、甲氧氯普胺、东莨菪碱、吩噻嗪等，根据呕吐严重程度不同可以选择单药、二联、三联或四联方案。呕吐发生率>30%的药物属于中度致吐风险，根据NCCN止呕指南，塞瑞替尼为中度致吐风险，需预防性止呕，药物建议选择5-HT_3_受体拮抗剂；克唑替尼、达拉非尼、Cabozantinib为中度致吐风险，但不建议预防性止呕，可根据实际情况选择止呕方案。使用止呕药物时应注意筛查与靶向药物的相互作用。

肺癌小分子靶向药物致便秘发生率方面，ALK抑制剂中克唑替尼、阿来替尼、恩沙替尼发生率较高，分别为37.3%、31.7%和31.5%；塞瑞替尼、洛拉替尼、布格替尼发生率相对较低，分别为19.1%、17.5%和15.0%^[40]^。恩曲替尼、安奈克替尼在临床试验中的发生率在30%以上^[48]^。EGFR-TKIs致便秘的发生率相对较低，在10%左右^[39]^。便秘表现为排便困难和/或排便次数减少、粪便干硬，常用对症治疗药物为聚乙二醇、乳果糖等。

##### 3.2.1.3 药物性肝损伤（drug-induced liver injury, DILI）

DILI是肺癌小分子靶向药物的常见ADR，以肝酶和胆红素等实验室指标升高为主要判断依据。临床试验数据显示，吉非替尼^[49,50]^、厄洛替尼^[51]^、克唑替尼^[52-55]^、恩沙替尼^[56,57]^、赛沃替尼^[58]^、恩曲替尼^[48]^、瑞普替尼^[59]^、伯瑞替尼、Lazertinib（与埃万妥单抗联用）、依奉阿克、格索雷塞致DILI发生率均有超过30%的报道。

DILI的临床表现与其他性质的肝损伤相比通常无特异性，目前仍属于排他性诊断。DILI的诊断、评估和治疗可参考《中国药物性肝损伤诊治指南》^[60]^。各靶向药物药品说明书通常已对DILI情况下的停药/减量方案作出了明确推荐，此外也可参考FDA建议的临床试验停药标准，即满足以下任意条件即可考虑停药：（1）血清天冬氨酸氨基转移酶（aspartate aminotransferase, AST）或丙氨酸氨基转移酶（alanine aminotransferase, ALT）>8倍正常值上限（upper limit of normal, ULN）；（2）AST或ALT>5 ULN，持续2周；（3）AST或ALT>3 ULN，且总胆红素（total bilirubin, Tbil）>2 ULN或国际标准化比值（international normalized ratio, INR）>1.5；（4）AST或ALT>3 ULN，伴肝损伤症状。

##### 3.2.1.4 心脏毒性

靶向药物的心脏毒性易造成严重后果，应高度重视，早发现，早处置。QT间期延长、心动过缓、心肌收缩力改变等是肺癌小分子靶向药物引起的心脏毒性的主要表现，患者可表现为疲劳、头晕、心悸、晕厥、癫痫或不明原因意识丧失等。奥希替尼^[61]^、克唑替尼^[62]^、塞瑞替尼^[63]^等肺癌小分子靶向药物在临床试验中曾观察到心脏ADR的发生，附表4列出这些药物心脏毒性的表现及全级别心脏不良反应的发生情况。根据药品说明书及III期临床试验中对心脏毒性的监测措施^[64]^，本文在肺癌小分子靶向药物监护路径（附表8，http://www.lungca.org/files/2024s64-s8.pdf）中给出心脏毒性监测指引。处理原则可参考《酪氨酸激酶抑制剂心血管毒性药学综合管理中国专家共识》^[65]^，并注意排查可致心脏毒性药物的合并使用。

##### 3.2.1.5 ILD

肺癌小分子靶向药物致ILD是发生率较高的致命性ADR，多种靶向药物临床试验中曾观察到发生ILD后死亡的病例^[66-68]^。肺癌小分子靶向药物所致ILD可表现为急性、亚急性起病，也可表现为慢性隐匿起病。患者表现为咳嗽（干咳为主）、呼吸困难、发热等，不及时处理可致双肺纤维化和低氧血症型呼吸衰竭。不同靶向药物引起ILD的发生时间不同，如吉非替尼所致ILD大多发生在用药后4周内^[69]^，奥希替尼所致ILD发生的中位时间约为用药后2.8个月。一旦发生或怀疑ILD，应立即停用肺癌小分子靶向药物，若有引起或加重ILD的合并用药（如博来霉素、胺碘酮等），应换用其他对ILD无影响的药物。对于确诊或高度怀疑靶向药物相关性ILD的患者，应立即给予糖皮质激素治疗。详细处置方案可参考《抗肿瘤药物相关间质性肺病诊治专家共识》^[70]^。

##### 3.2.1.6 其他ADR

肺癌小分子靶向药物还可引起多种其他ADR，如高脂血症、高血压、高血糖、神经毒性、视力障碍、手足综合征、外周水肿等，这些ADR多为药物特异性。如洛拉替尼导致的高脂血症是其最常见的ADR，在其他靶向药物中并不常见，其处理可参考《洛拉替尼特殊不良反应管理中国专家共识》^[71]^。布格替尼、维莫非尼、塞普替尼、Cabozantinib致高血压较常见。多个靶向ALK、ROS1重排的药物在临床试验中观察到血糖升高。洛拉替尼、恩曲替尼、瑞普替尼、拉罗替尼的临床试验中曾报告了各种认知受损症状^[48,53,59,72]^。洛拉替尼、瑞普替尼致周围神经病发生率分别为33.6%^[40]^和47.0%^[59]^。克唑替尼可导致视力障碍，且发生率较高，一般在给药开始2周内出现^[62]^。恩曲替尼报告了42.9%的手足综合征发生率^[48]^。MET-TKIs较为常见的ADR为外周水肿，II期临床试验中亚洲人群水肿发生率方面，赛沃替尼为54.0%^[58]^、谷美替尼为80.0%^[73]^、伯瑞替尼为56.6%^[74]^、特泊替尼为47.4%^[75]^、瑞普替尼为31.1%^[76]^。恩沙替尼致咳嗽发生率为30.8%^[40]^。阿美替尼致血肌酸激酶升高发生率为35.5%^[77]^。贝福替尼致血小板减少发生率为61.0%、肺栓塞发生率为5.8%、静脉血栓栓塞和血栓事件发生率为12.2%。瑞齐替尼致白细胞减少发生率为39.9%，血小板减少为30.2%。应用以上靶向药物时应特别关注其药物特异性ADR。

#### 3.2.2 ADR监测路径

肺癌小分子靶向药物所致ADR类型及发生率差异较大，此前尚无针对肺癌小分子靶向药物的不良反应监测路径，既往不良反应监测工作难以全面系统展开。因此，本共识根据ADR发生特点，以药物为中心建立了肺癌小分子靶向药物ADR监测路径（附表8），包括基线检查项目、复查项目及频次和需监测的症状，临床实践中应根据各靶向药物的监测路径进行个体化监测。

推荐意见8：根据附表8中ADR监测路径中的基线检查项目、复查项目及频次和需监测的症状，为患者制定疗效和安全性监测计划，主动随访，及时对症处理和剂量调整。（推荐级别：1类）

## 4 提高患者依从性

肺癌小分子靶向药物一般由患者遵医嘱在院外服用，因此患者的用药依从性对靶向药物的疗效和安全性影响非常明显。一项针对接受TKIs治疗的NSCLC患者的回顾性研究^[78]^结果表明，约50%的患者在启动治疗60天时停用TKIs，仅有38.3%的患者在120天后仍坚持服用TKIs，患者依从性差导致的疗效或安全性问题可能最终导致患者转换治疗药物、放弃治疗或死亡。药学监护工作可通过以下方式提高使用肺癌小分子靶向药物患者的依从性：（1）提供疾病和药物的信息支持，让患者了解靶向治疗的作用机制、预期疗效，加强用药教育，正确应对漏服和不良反应；（2）邀请患者参与治疗决策^[79]^；（3）制定小分子靶向药物的疗效、安全性随访计划，及时调整用药方案，处理不良反应。安全性随访的同时评估患者依从性和生存质量，并针对性制定管理策略。依从性可使用药物持有率（medication possession ratio, MPR）和服药天数比（proportion of days covered, PDC）^[80]^等进行评估，生存质量评分工具可选用美国东部肿瘤协作组体能状态（Eastern Cooperative Oncology Group performance status, ECOG PS）评分、卡氏功能状态（Karnofsky performance status, KPS）评分、欧洲癌症研究与治疗组织生活质量问卷-核心30条目（European Organization for Research and Treatment of Cancer Quality of Life Questionnaire-Core 30, QLQ-C30）^[81]^。

用药教育可提高患者用药知识水平，提高用药依从性，降低用药错误发生率。用药教育工作内容应包括：用药目的、儿童/孕妇/哺乳期用药、用法用量、用药期间应注意的情况、定期监测项目、DDI、ADR、贮存条件，并针对患者具体情况进行针对性教育。

推荐意见9：依从性差可显著影响肺癌小分子靶向药物治疗结局，应采取以下措施提高患者依从性：提供疾病和药物的信息支持，邀请患者参与治疗决策，制定小分子靶向药物的疗效和安全性随访计划，及时调整用药方案，处理ADR。此外，应常规开展肺癌小分子靶向药物用药教育工作。（推荐级别：1类）

## 5 总结与展望

我国有数十万驱动基因阳性NSCLC患者，小分子靶向药物在此类患者的治疗中占据重要地位。提高肺癌小分子靶向药物的合理用药水平、完善疗效和ADR监测质量和效率，是当前肺癌诊疗工作面临的重要课题。本共识针对国内外上市的43种肺癌小分子靶向药物或药物组合，就药物选择、剂量调整、疗效及ADR监测等问题形成推荐意见，指导临床用药，保障肺癌患者的药物治疗效果和用药安全性。此外，为提高查阅便利性、信息收容量和更新频率，我们正积极推进共识相关微信小程序等在线工具的开发，以便在未来进一步助力肺癌诊疗工作，惠及更多肺癌患者。

**编写委员会成员名单**

**组织编写机构：**广东省女医师协会肺癌专业委员会

**顾问：**吴一龙（广东省肺癌研究所，广东省人民医院，广东省医学科学院）

**专家组组长：**唐可京（中山大学附属第一医院）

**执笔：**赵丽岩（中山大学附属第一医院），唐可京（中山大学附属第一医院）

临床专家组（以姓氏拼音为序）：储天晴（上海交通大学附属胸科医院），崔久嵬（吉林大学第一医院），董晓荣（华中科技大学同济医学院附属协和医院），方文峰（中山大学肿瘤防治中心），胡洁（复旦大学附属中山医院），刘雨桃（中国医学科学院肿瘤医院），龙健婷（中山大学附属第一医院），涂海燕（广东省人民医院），汪斌超（广东省人民医院），邬麟（湖南省肿瘤医院），周承志（广州医科大学附属第一医院），周清（广东省人民医院），赵明芳（中国医科大学附属第一医院）

**药学专家组（以姓氏拼音为序）：**陈杰（中山大学附属第一医院），陈攀（中山大学附属第一医院），戴晓雁（甘肃省肿瘤医院），方罗（浙江省肿瘤医院），黄其春（广西医科大学附属肿瘤医院），黄红兵（中山大学肿瘤防治中心），简晓顺（广州医科大学附属肿瘤医院），孟珺（中国医学科学院肿瘤医院深圳医院），李国辉（中国医学科学院肿瘤医院），李晋奇（四川省人民医院），李亦蕾（南方医科大学南方医院），刘韬（中山大学肿瘤防治中心），刘继勇（复旦大学附属肿瘤医院），史琛（华中科技大学同济医学院附属协和医院），唐欲博（中山大学附属第一医院），伍俊妍（中山大学孙逸仙纪念医院），魏理（广州医科大学附属第一医院），肖洪涛（四川省肿瘤医院），钟诗龙（广东省人民医院），赵荣生（北京大学第三医院），赵丽岩（中山大学附属第一医院）

**秘书组：**曾嘉炜（中山大学附属第一医院），闫佳佳（中山大学附属第一医院）
